# Black soldier fly protein–based microencapsulation of lemongrass oil improves rumen fermentation efficiency and mitigates methane production *in vitro*

**DOI:** 10.14202/vetworld.2026.39-51

**Published:** 2026-01-06

**Authors:** Maharach Matra, Chaichana Suriyapha, Gamonmas Dagaew, Rittikeard Prachumchai, Srisan Phupaboon, Sukruthai Sommai, Theerachai Haitook, Sajee Kunhareang, Metha Wanapat

**Affiliations:** 1 Division of Animal Science, Department of Agricultural Technology, Faculty of Technology, Mahasarakham University, Maha Sarakham 44150, Thailand; 2 Animal Production Innovation and Management Division, Faculty of Natural Resources, Prince of Songkla University, Hat Yai Campus, Songkhla 90110, Thailand; 3 Nakhon Phanom Provincial Livestock Office, Department of Livestock Development, Nakhon Phanom 48000, Thailand; 4 Department of Agricultural Technology, Faculty of Science and Technology, Thammasat University, Pathum Thani 12120, Thailand; 5 Department of Biology, Faculty of Science, Mahasarakham University, Maha Sarakham 44150, Thailand; 6 Department of Animal Science, Faculty of Agricultural Technology, Buriram Rajabhat University, Buriram 31000, Thailand; 7 Department of Animal Science, Faculty of Agriculture, Khon Kaen University, Khon Kaen 40002, Thailand; 8 Tropical Feed Resources Research and Development Center, Department of Animal Science, Faculty of Agriculture, Khon Kaen University, Khon Kaen 40002, Thailand

**Keywords:** black soldier fly protein, climate-smart ruminant nutrition, essential oil microencapsulation, lemongrass oil, methane mitigation, rumen fermentation, sustainable feed additive, *in vitro* gas production

## Abstract

**Background and Aim:**

Essential oils (EOs) are promising natural modifiers of rumen fermentation and methane production; however, their volatility and rapid degradation limit their effectiveness. Microencapsulation can shield bioactive compounds and allow controlled release. Insect-derived proteins, especially from black soldier fly (BSF; *Hermetia illucens* L.), offer a sustainable and functional wall material, yet their use for rumen-targeted delivery remains unexplored. This study aimed to assess the effects of microencapsulated-lemongrass oil (M-LEO) using BSF protein as a biopolymer wall on gas kinetics, nutrient degradability, rumen fermentation parameters, microbial populations, and methane output *in vitro*.

**Materials and Methods:**

A completely randomized design was used with five dietary treatments containing M-LEO at 0, 2, 4, 6, and 8% of total dry matter (DM) substrate. *In vitro* rumen fermentation was performed using rumen fluid from Holstein-crossbred dairy cattle. Fermentation was measured at 12, 24, and 48 h for gas kinetics, *in vitro* dry matter degradability (IVDMD) and *in vitro* organic matter degradability (IVOMD), pH, ammonia-nitrogen (NH_3_-N), volatile fatty acids (VFAs), methane production, and microbial populations quantified by real-time polymerase chain reaction.

**Results:**

M-LEO showed high encapsulation efficiency (85.2%) and significant bioactive content. Supplementing with M-LEO notably improved gas production kinetics and nutrient degradability, with optimal effects at 6% of total DM. At this level, IVDMD and IVOMD increased by up to 11.5% and 10.5%, respectively. Total VFA and propionate concentrations rose significantly (p < 0.05), while acetate proportion and the acetate-to-propionate ratio decreased. Rumen pH and NH_3_-N levels stayed within optimal ranges and were unaffected by treatment. Methane production was substantially reduced, with decreases of up to 48.8% at 48 h compared to the control. Additionally, M-LEO boosted populations of key cellulolytic bacteria (*Fibrobacter succinogenes*, *Ruminococcus albus*, and *Ruminococcus flavefaciens*) and *Megasphaera elsdenii*, while significantly suppressing methanogenic archaea (*Methanobacteriales*).

**Conclusion:**

Microencapsulation of lemongrass oil with BSF protein effectively enhances rumen fermentation efficiency and significantly decreases methane emissions *in vitro*. This innovative insect-protein delivery system provides a sustainable and climate-friendly feed additive approach, deserving further validation *in vivo*.

## INTRODUCTION

Currently, a wide variety of plant-derived bioactive compounds (BCs), including phenolics, flavonoids, and especially essential oils (EOs), have been shown to influence rumen fermentation processes. As a result, plant-based extracts have gained significant interest as natural feed additives for ruminants [1]. EOs are complex mixtures of volatile, lipophilic secondary metabolites produced by plants and are responsible for their characteristic aroma and color [2]. These compounds are typically extracted using hydro-distillation, solvent extraction, or similar methods [3]. Due to their antimicrobial properties, EOs are increasingly recognized as natural alternatives to synthetic antibiotics in animal nutrition [4]. Furthermore, several EOs demonstrate strong antioxidant activity; for instance, EOs from clove, oregano, parsley, and tarragon have been shown to inhibit 2,2-diphenyl-1-picrylhydrazyl (DPPH) radicals by up to 50% [5]. Collectively, these properties enable EOs to affect key rumen fermentation traits that are vital for ruminant productivity and health [6].

Lemongrass (*Cymbopogon citratus* Stapf) is an important source of EOs and is widely cultivated in Central and South America, Africa, and tropical and subtropical parts of Asia [7]. Lemongrass EO shows broad-spectrum antimicrobial activity against yeasts, gram-positive, and gram-negative bacteria [8]. Dietary addition of lemongrass has been reported to boost nutrient breakdown and enhance rumen fermentation efficiency [9]. However, the practical use of EOs is limited by their high volatility and sensitivity to environmental factors like light, humidity, and temperature [10]. These physical and chemical properties pose significant challenges to the stability and effectiveness of EOs across various industrial applications [11]. To address these issues, microencapsulation technology is commonly used to protect EO bioactivity and allow controlled release under specific conditions [12].

Microencapsulation involves trapping small particles or droplets within a homogeneous or heterogeneous matrix or coating material to form microcapsules [13]. This technology enhances the handling, stability, and delivery efficiency of BCs [14]. The encapsulated core material is gradually released through the capsule wall, enabling sustained and controlled delivery. A wide variety of wall materials can be used, including natural polymers (e.g., cellulose, chitosan, and starch) and synthetic polymers (e.g., polyethylene, polyester, and nylon), depending on the desired properties of the final product [15]. Recently, insects have emerged as a promising alternative source of wall materials due to their high-quality protein content [16, 17]. In this study, proteins derived from the black soldier fly (BSF; *Hermetia illucens* L.) were chosen as a novel encapsulating material. BSF larvae contain 36%–46% crude protein with a favorable amino acid profile, along with substantial levels of saturated fatty acids and chitin (5%–8%) [18]. Selecting an effective wall material is crucial for spray-drying microencapsulation, as it directly impacts capsule stability and encapsulation efficiency. Importantly, BSF represents a circular bioresource capable of transforming organic waste into high-value protein, making it suitable not only for feed applications but also as an innovative and sustainable encapsulant for delivering bioactive compounds. Through regulated diffusion across the capsule wall, BCs can be released gradually, ensuring prolonged biological activity [19].

Plant-derived BC, especially EO, has been thoroughly studied for its ability to influence rumen fermentation and reduce enteric methane emissions. Many *in vitro* and *in vivo* studies show that EO can modify volatile fatty acid (VFA) profiles, inhibit methanogenic archaea, and enhance nutrient use in ruminants. However, the practical use of EO in ruminant diets remains inconsistent and often limited by their high volatility, quick breakdown in the rumen, low bioavailability, and antimicrobial effects that depend on dose and may harm beneficial rumen microbes. Although microencapsulation has become a promising method to improve the stability, handling, and controlled release of EO, most previous research has used traditional wall materials such as starch, maltodextrin, chitosan, or plant- and dairy-derived proteins. These materials can face issues related to cost, encapsulation efficiency, thermal stability during feed processing, or sustainability concerns.

Meanwhile, insect-derived proteins, especially those from BSF larvae, have gained recognition as sustainable feed ingredients due to their high protein content, favorable amino acid profile, and their role in circular bioeconomy systems. Despite these benefits, the potential of BSF protein as a functional wall material for microencapsulation of EO has not been thoroughly investigated. Currently, there is a noticeable lack of information on how insect-protein–based encapsulation systems affect the release of essential oil bioactive compounds in the rumen environment, their interactions with rumen microbial communities, and their subsequent impact on fermentation efficiency and methane production. Additionally, limited data exist on the best inclusion levels of microencapsulated EO using novel biopolymer walls that enhance fermentation benefits while reducing negative effects on rumen function. This knowledge gap hinders the development of scalable, climate-smart, and biologically efficient feed additives aimed at methane mitigation in ruminant production systems.

To address these gaps, this study aimed to evaluate the potential of BSF protein–based microencapsulation as a new delivery system for lemongrass essential oil in ruminant nutrition. Specifically, it sought to examine the effects of microencapsulated-lemongrass oil (M-LEO) on *in vitro* rumen gas kinetics, nutrient degradability, fermentation traits, rumen microbial populations, and methane emissions. Additionally, the research aimed to identify an optimal supplementation level that boosts rumen fermentation efficiency and shifts microbial pathways toward less methanogenesis without affecting rumen pH or nitrogen metabolism. By combining insect biotechnology with plant-based feed additives, this study provides mechanistic and applied insights into a sustainable, controlled release approach to enhance rumen function and reduce greenhouse gas emissions, supporting the development of climate-smart feeding strategies for ruminant livestock.

## MATERIALS AND METHODS

### Ethical approval

All experimental procedures involving animals were reviewed and approved by the Institutional Animal Care and Use Committee of Khon Kaen University (IACUC-KKU), Thailand (approval number: IACUC-KKU-86/66), and the Institute of Animals for Scientific Purpose Development (IAD), Thailand (approval number: U1-07387-2561). Animal handling, rumen fluid collection, and *in vitro* experimental procedures were conducted in strict accordance with national and international guidelines for the care and use of animals in research.

Rumen fluid was collected non-invasively from healthy dairy cattle maintained under standard management practices, causing minimal stress and discomfort to the animals. All experimental protocols adhered to the ethical principles outlined by the Animal Research: Reporting of *In Vivo* Experiments 2.0 guidelines for reporting animal research, ensuring transparency, animal welfare, and scientific validity throughout the study.

### Study period and location

This research was carried out from October 2024 to February 2025 at the Tropical Feed Resources Research and Development Center, Department of Animal Science, Faculty of Agriculture, Khon Kaen University, Khon Kaen, Thailand.

### Preparation of M-LEO

The microencapsulation process followed the method described by Phupaboon *et al*. [6]. A commercial BSF–derived protein extract was used as the wall material. The blending mixture consisted of a 1:1 (v/v) ratio of BSF protein extract suspension (20% w/v) and lemongrass oil extract (10% v/v), with Tween 80 (1% v/v) acting as an emulsifier. The uniform emulsion was processed using a spray-drying method (Büchi B-191 Mini Spray Dryer, Apeldoorn, Netherlands) under the following conditions: feed flow rate of 10 mL/min, drying airflow of 600 L/h, pressure drop of 0.75 bar, inlet temperature of 160°C, and outlet temperature of 90°C. The resulting microencapsulated powder was stored at −20°C until it was needed.

### Diet formulation and chemical composition analysis

The basal diet consisted of concentrate and roughage sources, as shown in Table 1. The concentrate was formulated with cassava chips, rice bran meal, palm kernel meal, soybean meal, urea, sulfur, mineral premix, and salt, providing a crude protein content of 14.6% on a dry matter (DM) basis. Rice straw was used as the roughage source, containing 2.4% crude protein, 78.9% neutral-detergent fiber (NDF), and 52.6% acid-detergent fiber (ADF).

**Table 1:** Dietary ingredients and nutritional composition.

Items	Concentrate	Rice straw	Black soldier fly extract	M-LEO
Ingredients (% as fed)	-	-	-	-
Cassava chip	54	-	-	-
Rice bran meal	17	-	-	-
Palm kernel meal	13	-	-	-
Soybean meal	10.5	-	-	-
Urea	2.5	-	-	-
Sulphur	1	-	-	-
Mineral premixed^1^	1	-	-	-
Salt	1	-	-	-
Chemical compositions	-	-	-	-
Dry matter (DM, %)	90.5	89.4	95.1	92.2
Organic matter	92.2	85.4	93.5	93.7
Crude protein	14.6	2.4	50	21.9
Neutral-detergent fiber	20.4	78.9	53.1	30.2
Acid-detergent fiber	12.6	52.6	38.9	23.5
Bioactive compounds	-	-	-	-
TPC (mg GAE/g DM)	-	-	-	1927.4
TFC (mg QUE/g DM)	-	-	-	789.2
Antioxidant capacities	-	-	-	-
ABTS (%)	-	-	-	12.8
DPPH (%)	-	-	-	78.1
FRAP (g TROE/g DM)	-	-	-	20.6
Encapsulation efficiency (%)	-	-	-	85.2

M-LEO = Microencapsulated-lemongrass oil, TPC = Total phenolic compounds, TFC = Total flavonoid compounds, ABTS = 2’-azino-bis (3-ethylbenzothiazoline-6-sulfonic acid), DPPH = 2, 2-diphenyl-1-picrylhydrazyl, FRAP = Ferric reducing antioxidant power.

1 Mineral premixed (contains per kg): vitamin A, 10,000,000 IU; vitamin D, 1,600,000 IU; vitamin E, 70,000 IU; Fe, 50 g; Mn, 40 g; Zn, 40 g; Cu, 10 g; I, 0.5 g; Se, 0.1 g; Co, 0.1 g.

Chemical analyses of concentrate, rice straw, and M-LEO were performed following AOAC methods [20] for DM (method no. 967.03), organic matter (OM; method no. 942.05), and crude protein (CP; method no. 984.13). Fiber fractions (NDF and ADF) were determined using an Ankom fiber analyzer (Ankom Technology Co., NY, USA) following the protocol of Van Soest *et al*. [21].

### Determination of BC and antioxidant capacity

M-LEO was analyzed for BC, including total phenolic and flavonoid contents, as well as antioxidant capacities. Total phenolic content was determined using the modified Folin–Ciocalteu method with gallic acid as the standard [22]. Total flavonoid content was quantified using a calibration curve generated from quercetin standards prepared by two-fold serial dilutions in ethanol [23]. Antioxidant activity was assessed using the DPPH radical scavenging assay [24], ABTS radical scavenging assay [25], and ferric reducing antioxidant power (FRAP) assay [26].

Encapsulation efficiency (EE) was determined using the equation: EE (%) = 100 × (total phenolic compounds extracted/total phenolic compounds encapsulated), as reported by Phupaboon *et al*. [27].

### *In vitro* experimental design and treatments

A completely randomized design was used with five dietary treatments, each involving M-LEO supplementation at 0, 2, 4, 6, and 8% of total DM substrate. Each treatment had three replicates and was tested at two incubation times, along with three blank replicates for each measured parameter.

### Animals, rumen fluid collection, and inoculum preparation

Rumen inoculum was collected from four Holstein-crossbred dairy cattle (Holstein Friesian × Thai native) with an average body weight of 500 ± 20 kg. The animals were fed rice straw *ad libitum* as roughage and a formulated concentrate diet to meet nutritional requirements for at least 14 days before rumen fluid collection. They were fed twice daily at 07:00 and 16:00 hours, with free access to mineral blocks and clean water. Rumen fluid was collected before the morning feeding using an orogastric tube connected to a vacuum pump, transferred into plastic flasks, and then placed in insulated Thermo Fisher Scientific bottles (USA) for immediate use.

### *In vitro* rumen incubation procedure

The *in vitro* incubation was performed using 50 mL bottles containing 0.5 g of DM substrate with a roughage-to-concentrate ratio of 60:40. M-LEO was added to the substrates based on treatment levels on a DM basis. Each bottle contained 40 mL of incubation medium, prepared from a buffer solution and rumen fluid at a 2:1 (v/v) ratio. All bottles were flushed with CO_2_, sealed with synthetic rubber stoppers and aluminum caps, and incubated at 39°C following the procedure of Menke and Steingass [28].

### Measurement of gas production kinetics

Gas production was measured using a 20 mL glass precision syringe at 1, 2, 4, 6, 8, 12, 24, 48, 72, and 96 h of incubation. Gas kinetic parameters were estimated with the nonlinear regression model of Ørskov and McDonald [29]:

Y = a + b (1 − e^-ct^),

where Y represents gas production (mL) at time t, a is gas production from the soluble fraction, b is gas production from the insoluble fraction, c is the fractional gas production rate, and t is incubation time (h).

### Rumen fermentation characteristics and methane analysis

Immediately after bottle opening at 12, 24, and 48 h, rumen pH was measured using a calibrated digital pH meter (HANNA Instruments HI 8424, Singapore). VFAs and ammonia-nitrogen (NH_2_-N) concentrations were determined after centrifuging filtered rumen fluid samples at 16,000 × *g* for 15 min. VFA analysis was performed using gas chromatography (HP6890, Hewlett-Packard Co., Ltd., NY, USA) following ether extraction, and the acetate-to-propionate ratio was calculated accordingly.

NH_2_-N concentration was measured spectrophotometrically using a LabAssay™ Ammonia kit (FUJIFILM Wako Pure Chemical Corp., Japan) with absorbance read at 630 nm. Methane production was analyzed with gas chromatography (GC-17A System, Shimadzu Co. Ltd., Kyoto, Japan) equipped with a thermal conductivity detector, following the method described by Pattra *et al*. [30].

### *In vitro* nutrient degradability

*In vitro* dry matter degradability (IVDMD) was measured by filtering incubation residues through pre-weighed Gooch crucibles and drying them at 100°C for 24 h. *In vitro* organic matter degradability (IVOMD) was evaluated after ashing the residues at 550°C for 4 h, following the method of Tilley and Terry [31].

### Microbial DNA extraction and quantitative polymerase chain reaction (PCR) analysis

Total genomic DNA was extracted from about 1 mL of rumen fluid using the QIAamp Fast DNA Stool Mini Kit (Qiagen, Hilden, Germany). DNA purity and concentration were measured with a Nanodrop spectrophotometer (Thermo Scientific, Waltham, MA, USA). Quantitative real-time PCR was conducted using Luna® Universal qPCR Master Mix with species-specific primers targeting key rumen bacteria and methanogens, including R*uminococcus albus*, *Ruminococcus flavefaciens*, *Fibrobacter succinogenes*, *Butyrivibrio fibrisolvens*, *Butyrivibrio proteoclasticus*, *Megasphaera elsdenii*, and *Methanobacteriales* [32–36]. Microbial populations were expressed as log_10_ gene copies per mL of rumen fluid DNA template [37].

### Statistical analysis

All data were analyzed using the general linear model procedure in SAS software (version 9.4; SAS Institute, Cary, NC, USA) under a completely randomized design. The statistical model was Y_ij_ = μ + M_i_ + ε_ij_, where μ represents the overall mean, M_i_ denotes the effect of M-LEO supplementation level, and ε^ij^ is the residual error. Treatment means were compared using Tukey’s test, and orthogonal polynomial contrasts (linear, quadratic, and cubic) were applied to assess responses to increasing M-LEO levels. Statistical significance was set at p < 0.05 and p < 0.01.

## RESULTS

### Nutritive composition and EE of M-LEO

M-LEO showed a DM content of 92.2%, with crude protein, NDF, and ADF contents of 21.9%, 30.2%, and 23.5%, respectively. The bioactive profile of M-LEO revealed high levels of total phenolic compounds (1927.4 mg GAE/g DM) and total flavonoid compounds (789.2 mg QUE/g DM). The EE, based on the recovery of total phenolic compounds within the encapsulated matrix, was 85.2%, demonstrating effective entrapment of bioactive substances (Table 1).

### Effects of M-LEO on gas kinetics and nutrient degradability

**Figure 1:**
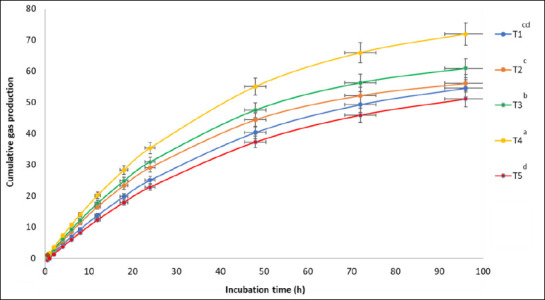
Cumulative gas production curves for the treatment substrates in an *in vitro* investigation (mL per 0.5 g dry matter of substrate). Treatments (n = 3; T1–T5) were supplemented with Microencapsulated-lemongrass oil at concentrations of 0%, 2%, 4%, 6%, and 8% of the total DM substrate. Error bar as mean ± standard deviation. ^a–d^ Means represented by different letters are significantly different at p < 0.05.

Supplementation with M-LEO significantly affected cumulative gas production (p < 0.01), as shown in Figure 1. Higher levels of M-LEO led to increased gas production from the immediately soluble fraction (a; p < 0.05), the insoluble fraction (b; p < 0.01), the gas production rate constant (c; p < 0.01), the potential extent of gas production (a + b; p < 0.01), and cumulative gas volume at 96 h (p < 0.01) compared to the control. Additionally, including M-LEO at 2–6% of the total DM substrate improved IVDMD and IVOMD at 12, 24, and 48 h, whereas a reduction was observed at the 8% inclusion level. The most significant improvements in degradability were seen at 48 h with 6% M-LEO supplementation, where IVDMD and IVOMD increased by 11.5% and 10.5%, respectively (Table 2).

**Table 2:** *In vitro* rumen gas kinetics and nutrient degradability after M-LEO supplementation.

Treatments	M-LEO	Gas kinetics^1^				Cumulative	IVDMD (% DM)			IVOMD (% DM)		
			
		*a*	*b*	*c*	*a* + *b*	gas^2^	12 h	24 h	48 h	12 h	24 h	48 h
1	0	-0.9^bc^	62.9^bc^	0.022^b^	62.0^bc^	55.6^cd^	51.0^b^	55.9^bc^	60.9^c^	57.9^b^	63.0^bc^	68.2^c^
2	2	-1.1^cd^	61.2^bc^	0.028^a^	60.1^bc^	58.1^c^	51.9^b^	57.9^ab^	64.0^b^	58.9^b^	65.0^ab^	71.2^b^
3	4	-0.6^b^	66.7^b^	0.027^a^	66.1^b^	62.8^b^	53.3^ab^	58.3^ab^	65.6^b^	60.1^ab^	65.4^ab^	72.8^b^
4	6	-0.3^a^	79.6^a^	0.025^ab^	79.3^a^	73.6^a^	56.6^a^	59.7^a^	68.8^a^	63.4^a^	67.0^a^	76.2^a^
5	8	-1.3^d^	60.0^c^	0.021^b^	58.8^c^	52.5^d^	50.6^b^	54.6^c^	59.5^c^	57.1^b^	61.7^c^	66.8^c^
SEM		0.1	0.45	0.01	0.46	0.34	0.32	0.26	0.24	0.33	0.27	0.23
Orthogonal polynomials											
Linear		<0.01	<0.01	0.27	<0.01	<0.01	0.01	0.01	<0.01	0.01	0.01	<0.01
Quadratic		0.02	<0.01	<0.01	<0.01	<0.01	0.33	0.7	0.91	0.35	0.79	0.74
Cubic		0.08	0.97	0.27	0.94	0.42	0.8	0.49	0.31	0.75	0.46	0.24

M-LEO = Microencapsulated-lemongrass oil (% of total DM substrate), IVDMD = *In vitro* dry matter degradability, IVOMD = *In vitro* organic matter degradability, SEM = Standard error of the mean.

1 Gas production kinetics, a = Gas production from the immediately soluble fraction (mL), b = Gas production from the insoluble fraction (mL), c = Gas production rate constant for the insoluble fraction (mL/h), a + b = Potential extent of gas production (mL).

2 Cumulative gasses at 96 h (mL per 0.5 g DM substrate).^a–d^ Means within the same column with different letters are significantly different at p < 0.05.

### Methane production

A significant reduction in methane production was observed following M-LEO supplementation at 12 h (p < 0.05; linear effect), 24 h (p < 0.05; quadratic effect), and 48 h (p < 0.01; linear effect), with the most notable effects occurring at supplementation levels of 6%–8% of total DM substrate. Compared to the control group, methane emissions decreased by 27.8%, 58.7%, and 51.2% at 12, 24, and 48 h, respectively, when M-LEO was included at 8% of total DM substrate (Table 3).

**Table 3:** *In vitro* rumen volatile fatty acids and methane production following M-LEO supplementation.

Treatments	M-LEO	VFA (mol/100 mL)			C_2_:C_3_	Total VFA (mmol/L)	Methane production (%)		
	
		C_2_	C_3_	C_4_			12 h	24 h	48 h
1	0	67.6^a^	22.0^b^	10.5	3.1^a^	103.8^b^	7.2^a^	6.3^a^	4.3^a^
2	2	66.5^ab^	23.0^ab^	10.6	2.9^ab^	108.9^ab^	6.9^ab^	6.0^ab^	3.6^b^
3	4	66.0^ab^	23.0^ab^	11.0	2.9^ab^	110.0^ab^	6.6^ab^	5.2^b^	2.8^c^
4	6	65.1^b^	24.8^a^	10.1	2.6^b^	115.8^a^	5.5^ab^	3.6^c^	2.2^d^
5	8	67.0^ab^	22.0^b^	11.0	3.05^a^	105.5^b^	5.2^b^	2.6^d^	2.1^d^
SEM		0.23	0.24	0.22	0.10	0.43	0.20	0.15	0.10
Orthogonal polynomials									
Linear		0.02	0.03	0.77	0.02	0.01	0.04	<0.01	<0.01
Quadratic		0.80	0.53	0.38	0.82	0.87	0.40	0.01	1.00
Cubic		0.72	0.39	0.57	0.49	0.38	0.73	0.48	0.72

M-LEO = Microencapsulated-lemongrass oil (% of total dry matter substrate), VFA = Volatile fatty acids, C_2_ = Acetate, C_3_ = Propionate, C_4_ = Butyrate, C_2_:C_3_ = Acetate-to-propionate ratio, SEM = Standard error of the mean. ^a–c^ Means within the same column with different letters are significantly different at p < 0.05.

**Table 4:** *In vitro* rumen pH and ammonia-nitrogen concentrations of M-LEO supplementation.

Treatments	M-LEO	pH			Ammonia-nitrogen (mg/dL)		
	
		12 h	24 h	48 h	12 h	24 h	48 h
1	0	6.94	6.90	6.88	16.0	16.8	18.2
2	2	6.93	6.94	6.92	16.8	17.6	18.4
3	4	6.93	6.91	6.89	16.9	17.7	18.5
4	6	6.91	6.89	6.87	16.9	17.7	18.5
5	8	6.92	6.90	6.88	16.8	17.8	18.6
SEM		0.03	0.04	0.04	0.15	0.16	0.12
Orthogonal polynomials							
Linear		0.20	0.57	0.58	0.05	0.19	0.24
Quadratic		0.59	0.23	0.20	0.13	0.36	0.46
Cubic		0.47	0.51	0.63	0.61	0.73	0.84

M-LEO = Microencapsulated-lemongrass oil (% of total dry matter substrate), SEM = Standard error of the mean.

### Modulation of rumen microbial populations

M-LEO supplementation positively affected rumen microbial dynamics by increasing the abundance of important cellulolytic bacteria, including *Fibrobacter succinogenes*, *R. albus*, and *R. flavefaciens* (p < 0.05; linear effects). Additionally, populations of *Megasphaera elsdenii* significantly rose at 24 h (p < 0.01) and 48 h (p < 0.05), while *Butyrivibrio fibrisolvens* (12 h; p < 0.01) and *Butyrivibrio proteoclasticus* (48 h; p < 0.05) were also boosted by M-LEO supplementation. Importantly, the abundance of *Methanobacteriales* (methanogens) was notably decreased at 12 h (p < 0.01), 24 h (p < 0.05), and 48 h (p < 0.01), especially at inclusion levels of 6%–8% of total DM substrate, indicating effective suppression of methanogenic archaea (Table 5).

**Table 5:** *In vitro* rumen microbial population after M-LEO supplementation.

Species (log copies/mL)	IT (h)	M-LEO					SEM	Orthogonal polynomials		
	
		0	2	4	6	8		L	Q	C
*Fibrobacter succinogenes*	12	8.5	8.5	8.7	8.8	8.7	0.10	0.08	0.70	0.86
	24	8.4^ab^	8.8^ab^	8.3^b^	8.9^a^	8.6^ab^	0.11	0.17	0.73	0.02
	48	8.6^b^	9.9^ab^	11.0^ab^	11.4^a^	9.3^ab^	0.24	0.02	0.52	0.87
*Ruminococcus albus*	12	9.2	9.5	9.3	9.7	9.4	0.13	0.19	0.79	0.31
	24	8.7^b^	9.1^ab^	9.1^ab^	9.5^a^	9.5^a^	0.11	0.03	0.90	0.37
	48	9.1^b^	9.3^ab^	9.4^ab^	9.8^a^	9.3^ab^	0.13	0.03	0.62	0.74
*Ruminococcus flavefaciens*	12	8.8	8.8	9.0	9.2	8.3	0.34	0.37	0.88	0.89
	24	7.9	9.0	9.4	9.5	7.6	0.21	0.06	0.38	0.90
	48	7.8^b^	8.9^ab^	9.7^a^	10.1^a^	8.7^ab^	0.22	0.01	0.42	0.95
*Megasphaera elsdenii*	12	8.6	9.1	9.1	9.4	8.9	0.14	0.05	0.74	0.54
	24	8.7^c^	9.0^bc^	9.3^ab^	9.5^a^	8.9^c^	0.09	<0.01	0.81	0.75
	48	9.1^b^	9.2^b^	9.4^ab^	9.9^a^	9.3^b^	0.05	0.01	0.23	0.77
*Butyrivibrio fibrisolvens*	12	8.2^b^	9.0^a^	9.1^a^	9.1^a^	8.9^a^	0.11	<0.01	0.03	0.39
	24	8.1	8.5	8.8	8.9	7.9	0.16	0.07	0.62	0.88
	48	7.7	8.6	8.5	8.6	8.4	0.16	0.10	0.26	0.46
*Butyrivibrio proteoclasticus*	12	9.9	10.0	10.0	10.1	9.9	0.09	0.29	0.78	0.71
	24	9.8	10.2	10.1	10.1	9.8	0.12	0.27	0.35	0.43
	48	9.7^b^	10.1^ab^	10.1^ab^	10.4^a^	9.9^ab^	0.12	0.02	0.65	0.33
*Methanobacteriales*	12	10.7^a^	10.2^b^	9.3^c^	8.3^d^	8.2^d^	0.09	<0.01	0.03	0.25
	24	9.1^a^	9.2^a^	8.3^ab^	7.8^b^	7.5^b^	0.16	0.01	0.32	0.24
	48	8.6^a^	8.2^ab^	7.7^bc^	7.2^c^	7.3^c^	0.13	<0.01	0.69	0.85

M-LEO = Microencapsulated-lemongrass oil (% of total dry matter substrate), IT = Incubation time, SEM = Standard error of mean, L = linear, Q = Quadratic, C = Cubic. ^a–d^ Means within the same row with different letters are significantly different at p < 0.05.

## DISCUSSION

### Role of microencapsulation and suitability of BSF protein as wall material

Microencapsulation is an advanced technique for protecting, stabilizing, and delivering bioactive substances across various sectors, including pharmaceuticals, cosmetics, and agriculture. By entrapping active compounds within a protective matrix and controlling their release, microencapsulation improves the functional effectiveness and stability of bioactive ingredients [38]. Protein–based encapsulants are especially appealing due to their favorable functional properties, such as film-forming ability, emulsification, and water retention. Additionally, chitin–protein complexes can control EO release by creating dense structural barriers that regulate diffusion and physically restrict microbial access to the encapsulated compounds [39].

BSF protein is a sustainable, eco-friendly encapsulating material, as BSF larvae efficiently convert organic waste into high-value protein with minimal resource use and significantly lower methane emissions than traditional livestock systems [40]. In this study, M-LEO achieved an EE of 85.2%, consistent with previous findings by Phupaboon *et al*. [6], who reported 84.7% using BSF protein with garlic oil. The microcapsules were mainly spherical to irregular in shape, with particle sizes from 12.6 to 19.2 μm, demonstrating successful encapsulation and structural stability.

### Functional characteristics of BSF–derived encapsulants

Using BSF–derived protein as a wall material offers clear benefits for stabilizing EO. BSF larvae are high in protein and contain significant amounts of chitin, making up about 7%–26% of DM [41]. Chitin, the second-most-abundant natural polymer, and its derivative, chitosan, are well-known biopolymers with applications in the food, medical, and agricultural fields [42]. Protein–based encapsulants also exhibit excellent functionality, including water retention, emulsification, gelation, and film formation, making them highly effective for encapsulating hydrophobic BC, such as EOs [43]. Therefore, using BSF–derived proteins as encapsulating materials offers a new and promising method for stabilizing EO designed for rumen-targeted delivery.

### Effects of M-LEO on gas production and nutrient degradability

Supplementation with M-LEO increased cumulative gas production, especially at a 6% inclusion level of total DM substrate. This effect may result from the combined nutritional benefits of M-LEO, where lemongrass oil provides readily available energy, and BSF protein adds additional crude protein. However, the reduction in gas production and nutrient degradability at the 8% inclusion level indicates that excessive supplementation might hinder rumen microbial activity and fermentation efficiency. Previous *in vitro* studies have also shown that EO can either positively influence rumen fermentation or change gas production depending on the dosage [44, 45].

At 12, 24, and 48 h of incubation, IVDMD and IVOMD were enhanced by M-LEO supplementation, with the highest improvement seen at the 6% inclusion level. The bioactive components of M-LEO, including phenolics, flavonoids, and essential oils, may stimulate rumen microbial activity and improve substrate utilization. Supporting this idea, Amin *et al*. [46] showed that microencapsulation effectively protects EO from rapid ruminal degradation. Consistently, microencapsulated products demonstrate lower disappearance rates than non-encapsulated oils during *in vitro* incubation, indicating longer-lasting bioactivity. Additionally, phenolics and flavonoids may have prebiotic-like effects by encouraging beneficial microbial growth, thus improving feed degradation efficiency [9]. Similar gains in nutrient degradability have been reported for microencapsulated BC from duckweed [47].

### Modulation of rumen fermentation pathways and methane mitigation

M-LEO supplementation significantly increased total VFA concentration and the molar proportion of propionate while decreasing the acetate proportion. This change in fermentation pattern may be due to the controlled, gradual release of lemongrass bioactives from the BSF protein matrix, which alters hydrogen-utilization pathways in the rumen. Increased propionate formation diverts hydrogen away from methanogenesis, thus reducing methane production [48, 49]. Similar increases in total VFA and propionate production have been reported by Hassan *et al*. [50], as well as by Suriyapha *et al*. [51] and Phupaboon *et al*. [52] using microencapsulated phytogenic compounds.

In the present study, methane production was significantly decreased at 12, 24, and 48 h following M-LEO supplementation. Suppressing ruminal methanogenesis promotes a metabolic shift from acetate-to-propionate synthesis, as propionate formation is thermodynamically favored over methane production when hydrogen availability is limited [53]. Consistent with these findings, Amin *et al*. [46] reported that microencapsulated cinnamon oil improved VFA profiles while lowering methane emissions.

### Rumen pH, nitrogen metabolism, and microbial stability

Rumen pH and ammonia-nitrogen (NH_2_-N) concentrations remained unaffected by M-LEO supplementation and stayed within optimal physiological ranges for microbial activity. The observed pH range (6.87–6.94) is ideal for fibrolytic bacteria and supports efficient fiber breakdown without increasing the risk of ruminal acidosis [54]. Stable pH conditions may also help promote favorable fermentation outcomes by preserving an optimal environment for microbial metabolism [55]. Similarly, NH_2_-N concentrations ranged from 16.0 to 18.6 mg/dL, which aligns with the optimal levels (15–30 mg/dL) needed to support maximum microbial protein synthesis and rumen fermentation efficiency [56].

### Influence of M-LEO on rumen microbial populations

Supplementation with M-LEO at 6% of total DM substrate significantly increased the abundance of key cellulolytic bacteria, including *F. succinogenes*, *R. albus*, and *R. flavefaciens*, as well as *M. elsdenii*. These effects are likely linked to the availability and sustained release of BC, which may promote beneficial microbial growth while preventing premature degradation of these compounds. Previous studies have shown that phenolics, flavonoids, and EO can boost overall bacterial abundance in the rumen [57]. Agarwal *et al*. [58] also reported increased *R. flavefaciens* populations following peppermint oil supplementation, although excessive inclusion levels may inhibit microbial growth.

Additionally, M-LEO increased populations of *B. fibrisolvens* and *B. proteoclasticus*, bacteria known to play key roles in biohydrogenation by converting unsaturated fatty acids into stearic acid [59, 60]. Importantly, the abundance of *Methanobacteriales* (methanogens) was significantly reduced as M-LEO inclusion increased. BC have antimicrobial properties that selectively inhibit methanogens or interfere with essential methanogenic pathways, thereby decreasing methane production during rumen fermentation [61, 62]. Similar decreases in methanogen populations have been reported after supplementation with microencapsulated phytogenic compounds derived from lemongrass and mangosteen peel [63].

## CONCLUSION

This study showed that M-LEO, using BSF protein as a new wall material, effectively improved *in vitro* rumen fermentation while significantly reducing methane production. Supplementing with M-LEO, especially at 6% of the total DM, led to notable improvements in gas kinetics, nutrient breakdown, and fermentation patterns. Specifically, *in vitro* DM and organic matter digestion increased by up to 11.5% and 10.5%, respectively, along with a significant rise in total VFAs and propionate production, and a decrease in acetate proportion. Importantly, methane emissions dropped by as much as 48.8%, along with a significant reduction in methanogenic archaea, while populations of key bacteria that break down cellulose and produce propionate increased. Rumen pH and ammonia-nitrogen levels stayed within healthy ranges, indicating that M-LEO did not disturb rumen stability.

The findings emphasize the potential of BSF protein–based microencapsulation as a sustainable and effective delivery system for EO in ruminant nutrition. By allowing controlled release of BC, M-LEO provides a promising feed additive strategy to enhance feed utilization efficiency and reduce enteric methane emissions, supporting climate-smart, environmentally responsible livestock production. Using insect-derived proteins also aligns with circular bioeconomy principles by transforming organic waste into high-value functional ingredients.

A key strength of this study is its innovative combination of insect biotechnology and phytogenic feed additives. Using BSF protein as an encapsulating material offers a new, sustainable alternative to traditional wall materials. Additionally, the thorough evaluation of fermentation kinetics, nutrient degradability, microbial populations, and methane production provides solid mechanistic insight into how M-LEO works.

The main limitation of this study is its *in vitro* design, which may not fully reflect the complexity of *in vivo* rumen processes, animal responses, or long-term adaptation of the rumen microbiome. Additionally, the economic viability and large-scale production aspects of BSF protein–based microencapsulation were not assessed.

Future research should focus on validating these findings *in vivo* to assess animal performance, feed efficiency, methane emissions, and potential impacts on product quality. Long-term feeding trials are necessary to determine optimal inclusion levels and evaluate microbial adaptation over time. Additionally, techno-economic analyses and life-cycle assessments would be useful to support the commercial adoption of this encapsulation strategy.

In conclusion, BSF protein–based M-LEO is a new, sustainable, and effective method for influencing rumen fermentation and reducing methane emissions. This approach holds significant potential for developing next-generation feed additives that promote productive, environmentally friendly ruminant production systems.

## DATA AVAILABILITY

All the generated data are included in the manuscript.

## AUTHORS’ CONTRIBUTIONS

MM, SP, SK, TH, and MW: Conceptualization and design of the study. MM, CS, GD, RP, SP, and SS: Sampling and chemical analysis. MM: Statistical analysis. MM, SK, and MW: Drafted and revised the manuscript. All authors have read and approved the final version of the manuscript.
